# Efficacy and safety of myrrh in patients with incomplete abortion: a randomized, double-blind, placebo-controlled clinical study

**DOI:** 10.1186/s12906-020-02946-z

**Published:** 2020-05-12

**Authors:** Homeira Vafaei, Sara Ajdari, Kamran Hessami, Ayda Hosseinkhani, Leila Foroughinia, Nasrin Asadi, Azam Faraji, Sepideh Abolhasanzadeh, Khadije Bazrafshan, Shohreh Roozmeh

**Affiliations:** 1 Maternal-Fetal Medicine Research Center, Shiraz University of Medical Sciences, Shiraz, Iran; 2 Obstetrics and Gynecology Department, Shiraz University of Medical Sciences, Shiraz, Iran; 3 Student Research Committee, Shiraz University of Medical Sciences, Shiraz, Iran; 4 Research Center for Traditional Medicine and History of Medicine, Shiraz University of Medical Sciences, Shiraz, Iran; 5 Box Hill Hospital, FRANZCOG Eastern Health, Victoria, Australia; 6 Maternal- Fetal Medicine (Perinatology), Hafez Hospital, Chamran Ave, Shiraz, Iran; 7 Department of Radiology, Shiraz University of Medical Sciences, Shiraz, Iran

**Keywords:** Myrrh, Incomplete abortion, Herbal medicine, Commiphora myrrha

## Abstract

**Background:**

Myrrh (Commiphora myrrha (Nees) Engl.) has a long history of traditional use as a herbal medicine for different purposes. In ancient traditional Persian manuscripts, it has been noted that myrrh may act as uterine stimulant and probably cause complete abortion. However, there is no evidence to verify this comment. Therefore, the current study was carried out to evaluate the efficacy and safety of Myrrh in the treatment of incomplete abortion.

**Materials and methods:**

In a randomized double-blinded placebo controlled clinical trial, 80 patients with ultrasound-documented retained products of conception (RPOC) were assigned to receive capsules containing 500 mg of Myrrh oleo-gum-resin or a placebo three times a day for 2 weeks. The existence of the retained tissue and its size were evaluated by ultrasound examination at the beginning and end of the study.

**Results:**

After 2 weeks, the mean diameter of the RPOC in the Myrrh group was significantly reduced compared with the placebo group (*P* < 0.001). Meanwhile, the rate of successful complete abortion was 82.9% in the intervention group and 54.3% in the placebo group (*P* = 0.01). The patients in both groups reported no serious drug-related adverse effects.

**Conclusion:**

This study shows that Myrrh is effective and safe in the resolution of the RPOC and may be considered as an alternative option for treatment of patients with incomplete abortion. However, further studies on active compounds isolated from myrrh and their uterine stimulant effects are needed.

**Trial registration:**

This study was retrospectively registered at Iranian Registry of Clinical Trials (www.irct.ir) IRCT code: IRCT20140317017034N7.

## Introduction

Among documented pregnancies, it is estimated that 15–20% of them will end in miscarriage [1]. Abortion-related complications are still one of the most important causes of maternal mortality and morbidity especially in developing countries [2]. Incomplete abortion is a type of abortion where some products of conception have been expelled, while other parts of the fetus, placenta or membranes are still retained in the uterus. The World Health Organization reported that 87,000 women die each year due to the incomplete abortion and its complications in developing countries [3]. Integrated approach for the treatment of incomplete abortion involves surgical intervention, medication administration and expectant management. Surgical procedures such as dilation and curettage (D&C) and manual vacuum aspiration (MVA) are frequently used for the treatment of incomplete abortion and removal of retained products of conception (RPOC) [4].

Surgery is an invasive practice with potential side effects, especially in patients with RPOC who have increased vascularity on ultrasound imaging. In such cases D&C may lead to massive hemorrhage due to excessive myometrial invasion by the trophoblastic tissue or arteriovenous malformation [5].

A variety of more conservative approaches such as pharmacological treatment or expectant management have been developed over the past few decades in the hope of reducing unnecessary surgeries, while achieving lower maternal morbidity and mortality rates [6]. Prostaglandin analogs such as misoprostol, used for treating incomplete abortion, have been known to cause several side effects and are not recommended for patients with multiple previous caesarean sections, known allergy to misoprostol, mitral stenosis, marked hypertension, glaucoma, and severe asthma [7]. On the other hand, expectant management is an alternative option with no cost suggested for hemodynamically stable patients [8, 9]. Given the lack of evidence regarding the advantage of either approach in stable patients, the woman’s preference should be taken into account when deciding the treatment strategy [9].

Historically in Iran, many patients have used medicinal herbs and other alternative medicine due to their efficacy, safety, and lower costs in comparison to chemical agents. Traditional Persian medicine still plays a vital role in most areas of Iran and its neighboring countries. To this day, local herbal shops in Iran continue to provide medicinal herbs for minor and major illnesses [10].

Myrrh is a yellow nonvolatile oleo-gum-resin produced mostly by wounding the bark of shrubs belonging to the genus Commiphora (Burseraceae family). The genus Commiphora, which includes more than 200 plant species, is distributed within dry regions of northeastern Africa, southern Arabia and India [11]. True myrrh is known in Persian medicine as “Mur e Makki” and is sourced from the Commiphora myrrha (Nees) Engl [12, 13].

The analysis of myrrh composition revealed that it contains 7–17% volatile oil, 25–40% resin, 57–61% gum, and less than 4% impurities [14]. Furanosesquiterpenes are responsible for the characteristic odor of myrrh and its analgesic effect [15]. The approval of Myrrh as a flavoring substance by U.S. Food and Drug Administration have suggested its safety for human consumption [16].

It is known that Myrrh has antiproliferative, antioxidant, anti-inflammatory and antimicrobial effects and has also been used since ancient times in gynecological diseases, wounds, pain control, obesity, and coronary artery diseases [13, 17]. In a traditional Persian manuscript from the ninth century, Rhazes (Persian physician) mentioned that Myrrh is also useful in the treatment of allergic rhinitis [12].

Literature review reveals that the resinous extract of myrrh consists of terpenes (monoterpenes, diterpenes, sesquiterpenes, and triterpenes), steroids, and lignans [18, 19]. Limonene, a monocyclic monoterpene, elevate intracellular calcium concentration via opening voltage-gated calcium channels and cause myometrial contraction in the uterus of pregnant rats [20]. In addition, inducible nitric oxide synthase (iNOS), a key signaling pathway for quiescence and relaxation of the uterine muscle during pregnancy, acts via increasing NO production which is a key smooth muscle relaxant [21], while active compounds from myrrh have been shown to inhibit iNOS expression [22].

In an animal study, it was revealed that oral administration of herbal tincture of myrrh was more effective than oxytetracycline infusion into the uterus in expelling retained placenta, and it also improves the subsequent fertility rate of animals [23]. Moreover in a case report from Saudi Arabia, authors presented a 9-week pregnant woman who experienced severe abdominal pain due to uterine stimulation after using myrrh orally, but her condition resolved immediately after discontinuation of the Myrrh [24].

The above-mentioned in vitro and in vivo studies along with ethnomedical use of myrrh for treatment of retained placenta in different traditional systems [23, 25] confirmed that the constituents of myrrh possess uterine stimulant activities and can facilitate the expelling of the RPOC. However, to date, there has been no clinical trial documenting this effect of Myrrh and proving its effectiveness in the treatment of RPOC.

Considering that the incidence of RPOC is relatively high in abortions, introducing an effective treatment that prevents unnecessary hospitalization and makes health care more affordable would be significantly important in clinical practice. Therefore, we aimed to evaluate the efficacy and safety of Myrrh as an alternative option with potential advantages over expectant management in women with incomplete abortion. Furthermore, a color Doppler ultrasound scan was used to determine the exact size of the RPOC before and after treatment with Myrrh.

## Materials and methods

### Study design and ethical approval

This parallel randomized, double-blind, placebo controlled clinical trial was performed on 80 patients with incomplete abortion in the gynecology wards of Shahid Faghihi and Hazrat Zeinab hospitals (affiliated to Shiraz University of Medical Sciences).

The protocol was approved by the ethics committee of the Shiraz University of Medical Sciences (approval code: IR.SUMS.MED.REC.1398.005). This study was performed in accordance with the guidelines of the revised Declaration of Helsinki 2013. All women provided written informed consent before study entry. The trial is registered with the Iranian Registry of Clinical Trials (IRCT20140317017034N7).

### Inclusion and exclusion criteria for the study

The study was performed on 80 women who met the study criteria and had a confirmed incomplete abortion (spontaneous or induced). The sonographic diagnosis of RPOC was based on the appearance of a thickened endometrial echo complex or the presence of a heterogenous and hyperechoic endometrial mass consistent with RPOC, as has been described in previous studies [26].

Inclusion criteria: women aged 18–40, hemoglobin level > 9 g/dL, hemodynamically stable, gestational age equal to or smaller than 20 weeks of gestation based on last menstrual period which was confirmed by first trimester ultrasound scan, RPOC with an anterior-posterior diameter 15–50 mm in ultrasound imaging, and body mass index (BMI) ≤ 30. All women were advised of the alternative option of surgical evacuation and/or misoprostol, the standard practice at our hospitals. Exclusion criteria were: fever, pelvic tenderness, signs of infection in vaginal examination, any history of chronic medical diseases (such as hypertension, diabetes mellitus, coagulopathy, and etc.). Anterior-posterior diameter was selected between 15 and 50 mm, because previous clinical trials report favorable outcomes after expectant management of RPOC in this range of size [27, 28]. So, we aimed to compare outcomes of Myrrh therapy with those who received placebo in patients with RPOC ranging from 15 to 50 mm.

### Randomization and study groups

The study population was randomized into two groups by using computer-generated random numbers (www.randomizer.org, an online randomization tool). Only the principal investigator had the ability to access random codes in order to discontinue the treatment if any serious reactions occurred. One group (*n* = 40) received capsules containing 500 mg of Myrrh three times a day for 2 weeks. The other group (n = 40) received placebo capsules containing 500 mg of cornstarch for the same period of time. Both placebo and Myrrh capsules were made by our research pharmacist with the same physical appearance and packed in similar envelopes. All the study subjects, researchers who administered medication (gynecologist), the statistical analyzer, and radiologist were blinded to the type of drug allocated to patients.

### Plant material

Gum samples were purchased from a local herbal market at Shiraz, Iran. The samples were authenticated in the Faculty of Pharmacy at Shiraz University of Medical Sciences. When confirmed as Commiphora myrrha (Nees) Engl., oleo-gum-resin, voucher specimens (voucher number: PM 1263) were deposited in the herbarium. Samples were then powdered and the capsules were prepared. Each capsule contained 500 mg of gum. Due to the lack of evidence on the optimal dose of myrrh, the Myrrh capsule dosage was determined from the stated recommended doses and intervals in traditional Persian medical manuscripts.

### Outcome measurements

Each patient was given an appointment to return to the hospital after 2 weeks to be evaluated regarding the status of their RPOC by ultrasound examination. After explaining myrrh-related side effects as well as signs and symptoms of endometritis (as a complication of incomplete abortion), patients were contacted by telephone follow-ups every 2 days to assure treatment compliance and screening for any adverse events by asking open-ended questions. Also, the patients were asked not to use any other natural products or drugs during the study period (except for acetaminophen, as needed). A transvaginal color Doppler ultrasound was performed in our tertiary care center by one experienced radiologist (to eliminate inter-observer variability) at baseline and 2 weeks after treatment to assess the anterior-posterior diameter of RPOC and to detect the potential complications occurred in the uterus and adnexa. Ultrasound scans were acquired using a GE Voluson E6 (GE Medical Systems, Austria). After magnifying the image to fill at least two-thirds of the sonogram screen, the maximum distance across the borders of RPOC was measured as an anterior-posterior diameter in a longitudinal plane. Each measurement was taken twice with a 5-min interval and the average values were used in analysis. A transvaginal color Doppler ultrasound has been shown to have a 88% sensitivity and 68% specificity in diagnosis of RPOC [29]. Furthermore, the decreased measures of AP-diameter of RPOC has been shown to be an indicator of complete spontaneous resolution [30].

As a secondary outcome measure, the rate of complete abortion was identified for both groups at the end of study, which is defined as an empty uterine cavity with no visual sign of RPOC in the ultrasound imaging of a woman with conclusive evidence of RPOC in her previous scans [30].

### Statistical analysis and sample size calculation

Due to the lack of previous studies, the strategy of sample size calculation was based on a pilot experiment conducted in our center on 10 participants who showed a mean difference of − 6.7 ± 10 mm in AP diameter of RPOC after administration of myrrh. Then, by entering these pilot results in a statistical tool named “Sample Size Calculator for Comparing Two Independent Means” [31], sample size in each group was calculated as 35 patients for each group based on 80% power and an error of 5% (*P* < 0.05, two-sided). To compensate for possible dropouts, we recruited 40 patients in each group.

Statistical analysis was performed using SPSS (v 18; SPSS, Inc., Chicago, IL). Descriptive statistics were reported as mean ± SD for continuous variables and frequency (percentage) for categorical variables, respectively. After assessing the normality of data with Kolmogorov-Smirnov test, independent samples t-test and Chi-square for baseline variables and analysis of variance tests for outcome measurements were used in the statistical analysis. *P*-value < 0.05 was considered significant.

## Results

**Fig. 1:**
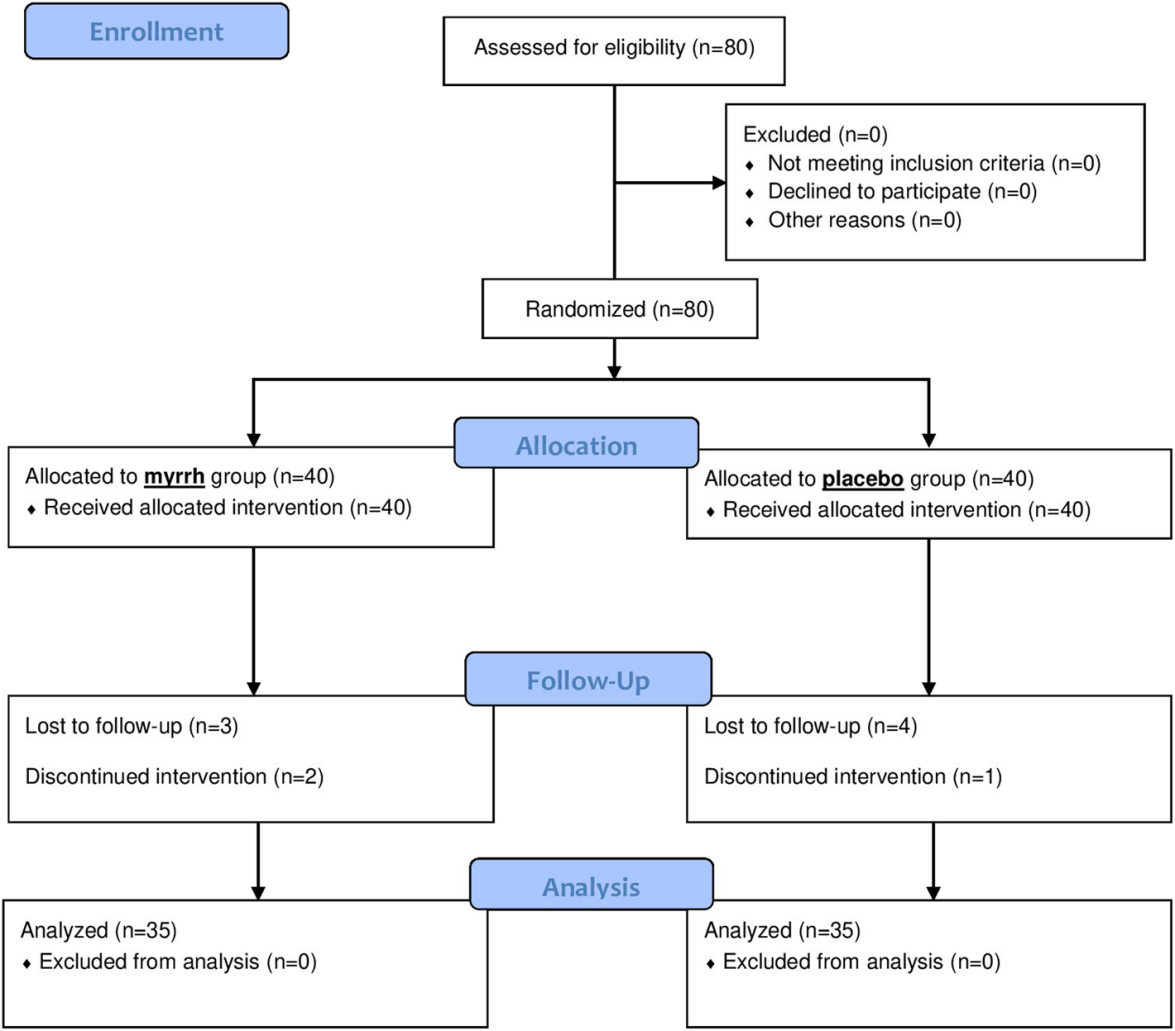
CONSORT flowchart of study

Eighty patients entered the study from May 2019 to August 2019. Three patients from the Myrrh group and 4 patients from the placebo group were lost to follow and did not respond the telephone calls. Two patients from the intervention group discontinued intervention due to personal reasons (contrary advice from a family member) and one patient from the placebo group discontinued the study due to signs and symptoms of pelvic inflammatory disease during the study necessitating the surgical evacuation of RPOC. Thirty-five patients in each of the Myrrh and placebo groups completed the trial. A flowchart of the study is shown in the Fig. 1.

According to the results of Table 1, there were no significant differences between the two groups in the baseline characteristics such as maternal age, gestational age (at the time of documenting incomplete abortion) and obstetrical history (gravidity, parity and abortions). The mean interval between abortion and starting intervention was similar for both groups (15.30 ± 9.20 days vs 16.00 ± 10.90 days, *P* = 0.772).

**Table 1:** Baseline characteristics of myrrh and placebo groups

Variables	Myrrh group (*n* = 35)	Placebo group (n = 35)	P-value*
Age (years)	31.2 ± 5.6	30.4 ± 6.2	0.573
Gestational age (weeks)	9.9 ± 3.6	9.6 ± 3.7	0.770
Gravidity	2.9 ± 1.5	2.4 ± 1.4	0.629
Parity	1.3 ± 1.2	1.1 ± 1.0	0.560
Abortion	1.5 ± 0.9	1.3 ± 0.6	0.403
Days since abortion	15.3 ± 9.2	16.0 ± 10.9	0.772

*Independent sample t-test

Data presented as mean ± SD

There was no significant difference in anterior-posterior diameter of RPOC in Myrrh and placebo groups before the intervention (*P* = 0.773). According to the ultrasound findings at the end of the study (week-2), the mean anterior-posterior diameter of RPOC in the Myrrh group was 4.6 mm (±1.0 mm) and in the placebo group it was 11.3 mm (±1.3 mm), which were significantly different (*P* < 0.001) (Table 2).

**Table 2:** Ultrasound measurement of retained products of conception size before and after treatment.

Ultrasound before and after intervention	Myrrh group (n = 35)	Placebo group (n = 35)	P-value*
Size of RPOC at week 0 (mm)	28.2 ± 13.5	27.4 ± 8.2	0.773
Size of RPOC at week 2 (mm)	4.6 ± 1.0	11.3 ± 1.3	< 0.001

*Independent sample t-test

Data presented as mean ± SD.

*RPOC*: Retained products of conception, mm: millimeters.

The rate of successful complete abortion, defined as no RPOC found in ultrasound examination at the end of study, was 82.9% in intervention group and 54.3% in placebo group (*P* = 0.01) (Table 3). No serious adverse drug reactions were observed or reported by study subjects.

**Table 3:** Ultrasound presence of RPOC after treatment

Ultrasound findings after 2 weeks	Myrrh group (n = 35)	Placebo group (*n* = 35)	P-value*
Presence of RPOC	6 (17.1%)	16 (45.7%)	0.01
Absence of RPOC	29 (82.9%)	19 (54.3%)	

*Chi-square test

Data presented as frequency (percentage)

RPOC: retained products of conception

## Discussion

The results suggest that use of Myrrh as a medication is an effective and safe therapeutic option in the management of incomplete abortion. Moreover, the results of ultrasound examination showed a significant decrease in the size of RPOC after treatment with Myrrh. To the best of our knowledge, this is the first study evaluating the efficacy and safety of Myrrh in patients with RPOC after abortion.

Findings of the current study showed that the complete abortion rate was 82.9% for the intervention group. Also, the current study suggests that Myrrh can be used as an alternative and safe therapeutic option in patients who are not candidates for surgical intervention or misoprostol therapy.

The exact mechanism by which Myrrh acts in the treatment of incomplete abortion is not absolutely clear. However, the most reasonable mechanism of action appears to be attributable to the uterine stimulant effect of Myrrh resin resulting in the expulsion of RPOC [32–34], and this is the reason that previous researchers proposed emmenagogue and abortifacient activities for myrrh and suggested myrrh not be administered to pregnant women [35, 36].

The authors of the current study could not find a clinical trial on the efficacy of myrrh consumption in patients with RPOC in the literature, but earlier in vitro and in vivo studies have recommended that different active compounds from myrrh could stimulate smooth muscle contraction, possibly by opening voltage-gated calcium channels [20] and inhibiting NO production [22]. Additionally, in a study on anti-ulcer activity of myrrh in rats, a prostaglandin-stimulant effect was noted for myrrh [37]. Prostaglandin stimulation is another possible mechanism leading to uterine contraction and expelling of RPOC.

It is well understood that long-lasting RPOC serves as a nidus for infection in the uterus and is one of the causes of postpartum endometritis or hemorrhage [38]. In our study, none of the participants in the intervention group developed endometritis, and it seems that myrrh has the potential to prevent endometritis as a complication of RPOC by its antimicrobial activity. Its antimicrobial effects seem to be hinged to a variety of mechanisms such as increased number of leukocytes, triggered phagocytic activity, and pro-inflammatory responses [32, 39].

In the scientific literature, there have only been a few clinical trials conducted to investigate the efficacy of Myrrh in different diseases. In a study by Sheir et al., Myrrh was administered at a dose of 10 mg/kg per day for three successive days to patients with schistosomiasis [40]. Their results demonstrated that treatment with myrrh led to 91.7% cure rate. This herb was well tolerated and caused less adverse reactions compared to praziquantel.

The analgesic effect of Myrrh has been studied in 184 patients in Italy [41]. The authors indicated that pain scores significantly decreased in male participants in the study after administration of 400 mg/day of Myrrh for 20 days, whereas, the alleviation of lower back pain and fever-dependent pain was brought about by only 200 mg/day of Myrrh in the female population [41].

Also in the clinical studies by Mansour et al. [42] and El-Sherbiny et al. [43], it was found that Myrrh is an effective and safe alternative medicine in the treatment of recurrent aphthous stomatitis and Trichomoniasis vaginalis infection, respectively.

The results of in vitro studies showed that extracts and compounds from myrrh could be used as novel preventive and therapeutic agents in human gynecologic cancers as well as prostate cancer [13, 44].

The safety of Myrrh, as we observed during the study period, may be yet another reasonable factor for its prescription in patients with incomplete abortion. A study by Langhorst et al. confirms the safety and tolerability of the long term use of Myrrh (1200 mg/day) plus chamomile extract (840 mg/day) and coffee charcoal (600 mg/day) in patients with ulcerative colitis [45]. No serious side effects were reported in previous trials using myrrh on human subjects [40, 41]. However, a toxicological evaluation of Myrrh essential oil was conducted in mice and it was seen that lower doses of myrrh (1, 5, and 10 μL) did not cause skin inflammation, swelling, dermatitis, scabbing, and abrasions. The liver and kidney enzymes were also found within the normal range which was comparable to the control group. On the other hand, in acute toxicological analysis, subcutaneous injections of higher doses of myrrh (20, 40, and 80 μL) adverse hepatic, renal and allergic events were noted in animal models [46].

In summary, the current evidence does not indicate the superiority of either expectant care (no treatment) or other interventions for incomplete abortions. However, if a woman opts for expectant management, she should be fully informed regarding the higher rate of RPOC, and the need for unplanned surgical evacuation, blood transfusion, pelvic infection, etc. [9].

Our study has its own limitations. Firstly, the study sample size was relatively small and only 70 patients were analyzed at the end-point of study. Secondly, we used a double-blind comparison with a placebo to eliminate a “placebo effect” for Myrrh, while the patients in the control group were supposed to be treated expectantly receiving no drug, even a placebo. Another limitation was the dosage of Myrrh capsules for patients with RPOC. Due to the lack of prior research on this subject, dosage was decided on an empirical basis obtained from traditional manuscripts.

## Conclusion

Myrrh, a well-known herbal medicine, adopted from traditional medicine textbooks, seems to be a safe and efficient treatment in women with RPOC. Future well-designed studies with larger sample size are required to verify our findings. Although the main constituents of Myrrh are known, it is not exactly clear which one acts as uterine stimulant agent resulting in complete abortion. Therefore, future investigations are recommended to determine the mechanism of action by which Myrrh causes uterine stimulation and reduces the size of RPOC.

## Data Availability

The datasets analyzed during the current clinical trial are available from the corresponding author on reasonable request.

## Abbreviations

D&C: Dilation and curettage
MVA: Manual vacuum aspiration.
RPOC: Retained products of conception.
iNOS: inducible nitric oxide synthase.
NO: Nitric oxide.
